# The Association between Type and Intensity of Sport and Tobacco or Nicotine Use—A Cross-Sectional Study among Young Swiss Men

**DOI:** 10.3390/ijerph17228299

**Published:** 2020-11-10

**Authors:** Marine Gossin, Gerhard Gmel, Joseph Studer, Mathieu Saubade, Carole Clair

**Affiliations:** 1 Department of Training, Research and Innovation, Center for Primary Care and Public Health (Unisanté), University of Lausanne, 1010 Lausanne, Switzerland; carole.clair@unisante.ch; 2 Addiction Medicine, Department of Psychiatry, Lausanne University Hospital and University of Lausanne, 1011 Lausanne, Switzerland; gerhard.gmel@chuv.ch (G.G.); joseph.studer@chuv.ch (J.S.); 3 Research Department, Addiction Switzerland, 1001 Lausanne, Switzerland; 4 Centre for Addiction and Mental Health, Institute for Mental Health Policy Research, Toronto, ON M5S 2S1, Canada; 5 Department of Health and Social Sciences, University of the West of England, Bristol BS16 1QY, UK; 6 Swiss Olympic Medical Center, Division of Physical and Rehabilitation Medicine, University Hospital of Lausanne, 1011 Lausanne, Switzerland; mathieu.saubade@chuv.ch; 7 Department of Prevention and Public Health, Center for Primary Care and Public Health (Unisanté), University of Lausanne, 1010 Lausanne, Switzerland

**Keywords:** tobacco products, cigarette, snus, snuff, vaping, type of sport, intensity of sport

## Abstract

The objective of this study was to assess the association between tobacco/nicotine use and type and intensity of sport. Data were drawn from the second follow-up of the Cohort Study on Substance Use Risk Factors. Young Swiss men completed a questionnaire about tobacco/nicotine use (cigarette, vaping, snus, snuff), type and intensity of sport and other demographic and medical variables. Among the 5414 included participants (mean age 25.5), 3434 (63.4%) reported regularly practicing a sport. They had a lower rate of cigarette smoking (32.3%) compared with participants not practicing a sport (44.6%) but a higher rate of snus use (15.0% vs. 10.0%). In adjusted models, individual-sport participants were less likely to use snus and snuff (OR = 0.63, 95% CI = 0.51–0.77 and OR = 0.73, 95% CI = 0.61–0.88), compared with team-sport participants. The association was inversed for vaping users (OR = 1.54, 95% CI = 1.03–2.30). Furthermore, participants who practiced high-intensity sports had a lower likelihood to smoke cigarettes (OR = 0.63, 95% CI = 0.52–0.78) compared with low-intensity sports. Our findings suggest that type and intensity of sport are associated with tobacco/nicotine use. Youth who practice an individual sport are less likely to use snus or snuff and more likely to vape compared with a team sport. This could help better target smoking prevention in young people

## 1. Introduction

Noncommunicable diseases are increasing worldwide and lifestyle, including tobacco use and physical activity, is a major influencing factor. Tobacco is an important risk factor for cancers [1], cardiovascular diseases, respiratory diseases and many other pathologies [2]. Furthermore, smokeless tobacco products are also risk factors for cancer, especially pancreas cancer, fatal myocardial infarction and other diseases [3]. Tobacco use has also an environmental and economic impact [4]. The young population is especially at risk to initiate tobacco or nicotine use and is heavily targeted by the tobacco industry through different channels [5,6,7]. Advertisement for tobacco products is not allowed on television and radio in Switzerland but advertising posters are allowed depending on the state regulation. In every European country, the advertisement of tobacco products is prohibited, except for display channel advertisement, which is allowed in Germany and Bulgaria [8]. In 2016, data from Switzerland indicated that among those aged 15–25 years, 31.6% were smokers, which represented an increase over the last years [9].

Physical activity is an important cardio-vascular protective factor. According to the World Health Organization (WHO) in the Global Recommendations on Physical Activity for Health from 2010 [10], sedentary lifestyle is increasing in our society and is a risk factor for global mortality. The current recommendations for adults (18 to 64 years) is: at least 150 min weekly of moderate intensity physical activity or 75 min weekly of high intensity [10]. In 2012 in Switzerland, according to the Federal Office of Public Health, 74% of the population was correctly following these recommendations (65% in 2002) [11]. Prevalence of regular sport activity (sport activity at least once a week) in 2020 in Switzerland was 83% (78% in 2014) among young men 15–24 years old and 70% (72% in 2014) among those aged 25–34 years [12,13].

Smoking and physical activity are two of the main modifiable lifestyle risk factors for morbidity and mortality. A healthy lifestyle is very important and can have a large impact on reducing the global burden of noncommunicable diseases [14]. This is why health education should be an important part of education among young people, to implement healthy habits. Prevention of smoking and sedentary lifestyle is, thus, important to decrease the incidence of noncommunicable diseases. There are unclear and even conflicting results about the link between physical activity and tobacco use (including cigarette smoking and smokeless tobacco). Some studies found an inverse association between tobacco use and physical activity, suggesting that people who are physically active, are less likely to smoke [15,16,17]. Others found a positive association suggesting that people who are more physically active are more likely to smoke [18,19,20]. In a systematic review from Kaczynski et al., in 2005, including 50 studies, the authors found a global negative association between physical activity and smoking, with an attenuated effect for young people [17]. The variability of results between studies could be explained by the various criteria used to define physical activity (frequency, time, duration, intensity) and smoking (frequency, quantity). In another study performed in the USA among adolescents between 14 and 18 years old [21], the association between smoking and sport varied depending on the type of sport. The authors divided sports in 2 categories: sports that were negatively associated with smoking like racquet sports, running, swimming, soccer and sports that were positively associated with smoking like skating, bicycling, fighting sports and competitive wrestling. They suggested that the type of sport could be more important than its frequency or intensity. Furthermore, in a Finnish study among young men during their first day of military service [22], the authors found an association between snus use and type of sport and intensity of physical activity, with a positive dose-response. They also found a lower rate of cigarette smoking among young men who practiced high intensity sport compared with those who did not practice a sport. Studies suggest that people who participate in a team sport have a higher tobacco use than those who participate in an individual sport [22,23].

Young people are influenced by their peers and social relations. Playing a sport (with or without peers) or engaging in a physical activity can influence behaviors such as tobacco use. Furthermore, some tobacco products are culturally influenced, for example snus, which is very widespread among ice hockey players [22]. The association between tobacco use and type of sports among young Swiss men has not been studied. The aim of this study was to assess the association between tobacco use (snus, snuff, cigarette smoking) and use of vaping among young Swiss men and type and intensity of sport practiced. A better understanding of the relationship between type and intensity of sports and tobacco or nicotine use could help better target smoking prevention in young people.

## 2. Materials and Methods

### 2.1. Study Design

We did a cross-sectional analysis of the tobacco or nicotine use and type of sport among young Swiss men. We used data from the second follow-up of the Cohort Study on Substance Use Risk Factors (C-SURF) from 2016 to 2018, because questions on types of sport and intensity were not asked in the preceding waves. The aim of the C-SURF study is to analyze the substance use and lifestyle among young Swiss men. Participants were enrolled in the study at the army recruitment in Switzerland between 2010 and 2011, with a baseline questionnaire between 2010 and 2012, a first follow-up during 2012–2014 and a second between 2016 and 2018. Participants received a questionnaire in French or in German (online or hard copy) with questions about their lifestyle, health, socio-economic situation, family, education, sexuality and substance use and their answers were analyzed. All data were self-reported. The research protocol of the C-SURF study was approved by the Ethics Committee of the canton de Vaud (CER-VD, research protocol number: 15/07). More information on the C-SURF study is available on the website of the study www.c-surf.ch.

### 2.2. Participants

Participants were young Swiss men over 18 years old. They were enrolled in the study at the army recruitment centers in Lausanne (VD), Windisch (AG) and Mels (SG). In Switzerland, recruitment is in principle mandatory for men (with exceptions of around 2% due to severe medical conditions). This offers the unique opportunity to invite young men of all socioeconomic strata and independent of whether participants served in the army, provided civil service or were non-eligible for any service. Participants were informed about the study and asked to sign a consent form at the army centers. Baseline questionnaire (45–60 min) was sent by email or post within two weeks after recruitment and completed at home. Thus, although enrolment began in the recruitment centers, the study was completely independent of the army. The first follow-up questionnaire was sent 18 months after the baseline questionnaire and the second follow-up 3 years after the first follow-up (per email or per post). They received small gifts (voucher) for each completed questionnaire. In total, 7556 participants signed the informed consent and were included from 2010 to 2011 but 5987 participants answered the baseline questionnaire, 6020 answered the questionnaire of the first follow-up and of them 5516 answered the questionnaire of the second follow-up (73% of the participants who initially signed the consent). We only used data from the participants of the second follow-up as explained above. After excluding participants with missing values for the variables of interest in the second follow-up as well as participants who gave non-contributing answers regarding the type of sport, the final sample included 5414 participants.

### 2.3. Tobacco Use

The dependent variable of interest was tobacco or nicotine use subclassified as cigarette smoking, snus use, snuff use and vaping. Participants were asked if they smoked cigarettes or vaped in the last 30 days and if yes, how frequently. The possible answers were: daily, 5–6 days/week, 3–4 days/week, 1–2 days/week, 2–3 days/month, once a month or less, never. We took the last 30 days as a cut-off, which is commonly used in most studies for the actual cigarette smoking. Use was assessed in the last 12 months for snus and snuff. We first classified tobacco and nicotine use in three categories as follows:

- cigarettes and vaping: no use, occasional (5–6 days/week, 3–4 days/week, 1–2 days/week, 2–3 days/month, once a month or less), daily
- snus and snuff: no use, occasional (2–3 days/month, once a month or less), weekly (daily, 5–6 days/week, 3–4 days/week, 1–2 days/week).

For the logistic regression models, tobacco or nicotine use (cigarettes, snus, snuff, vaping) was dichotomized into no use and use.

### 2.4. Physical Activity, Type and Intensity of Sport

The independent variables were physical activity and type of sport. We asked the participants their physical activity level during the week in their studies/work/apprenticeship (low, moderate and high), if they regularly practiced a sport, which sport they practiced the most frequently and how often. They also indicated the frequency and duration of the sports activity. The questions were adapted from the literature [24,25]. The answer about the type of sport was a free text. We created a dichotomous variable: sport vs. no sport. We excluded the non-contributive answers. After collecting the data, we ordered the sports into two categories: team sport or individual sport, with validation by a sport and exercise medicine specialist (MS). We also used Mitchell’s classification to divide the sports into 9 categories (A1, A2, A3, B1, B2, B3, C1, C2, C3) according to the static component (1, 2 and 3, “estimated percent of maximal voluntary contraction (MVC) reached resulting in an increasing blood pressure load”) and dynamic component (A, B and C, “estimated percent of maximal oxygen uptake (Max O2) achieved resulting in an increasing cardiac output”) of each sport. They are divided into low static component (<20% MVC), moderate (20–50% MVC), high (>50% MVC) and low dynamic component (<40% Max O2), moderate (40–70% Max O2), high (>70% Max O2)) (Supplementary Table S1) [26]. Sports were also classified by their intensity with Metabolic Equivalent of Task (MET) per minute per week according to the Compendium of Physical Activities [27] and the duration and frequency reported. Cut-offs were chosen according to the WHO recommendations [28]:

- 675 MET/min per week: minimum weekly energy expenditure recommended
- 1350 MET/min per week: weekly energy expenditure recommended for additional benefits on health.

We separated the intensity of a sport into three categories: low (less than 675 MET/min per week), medium (between 675 MET/min per week and 1350 MET/min*week) and high (more than 1350 MET/min per week).

### 2.5. Covariates

Potential confounding variables were defined *a priori* based on the literature and were the following: age, linguistic region (French- or German-speaking), self-reported body mass index (BMI) (continuous), highest achieved education (primary schooling if ≤9 years, vocational schooling if >9 to 12 years, post-secondary schooling if 13 years or more), cannabis use in the last 12 months (no use, less than weekly, weekly) and illicit drug use in the last 12 months (no use, use). We also used alcohol consumption in the last 12 months as confounding factor with two markers: binge drinking (≥6 drinks on one occasion, at risk if ≥ once a month [29,30]) and at risk use (≥21 drinks/week [31]).

### 2.6. Statistical Analysis

Statistical analyses, including descriptive analyses and logistic regressions, were realized with STATA/IC 15.1. We used descriptive statistics to present demographic, sample characteristics of the studied population and tobacco/nicotine use with chi^2^ tests for categorical and binary variables and student *t*-test for continuous variables. We built a logistic regression model to assess the association between tobacco and nicotine use and types of sport (team-individual, according to Mitchell’s classification) and sport’s intensity, with further adjustment for potential pre-identified confounding variables. In secondary analyses, separate logistic models were built for each type of tobacco or nicotine use (cigarette smoking, snus and snuff use, vaping) separately and odds-ratio and 95% confidence intervals were calculated. For the logistic models, tobacco and nicotine use variables were used as binary variable (use vs. no use). We performed a stepwise adjustment: unadjusted model, model 1 adjusted for socio-demographic variables (age, highest achieved education, language) and BMI and model 2 adjusted for socio-demographic variables, BMI and other substance use (cannabis, alcohol at risk use and drug).

## 3. Results

In total, 5516 participants answered the second follow-up questionnaire. Of them, 83 participants were excluded because of missing values and 19 because of non-contributive answers about type of sport, giving a final sample of 5414 participants. There were no statistically significant differences between excluded and included participants according to age (*p* = 0.722), spoken language (*p* = 0.874), cigarette smoking (*p* = 0.631), snus use (*p* = 0.787) or BMI (*p* = 0.270). Participants with missing values had a lower achieved education (*p* < 0.001), were more likely to vape (*p* = 0.017) and more likely to use snuff (*p* = 0.048).

### 3.1. Sample Characteristics

Sample characteristics of participants are presented in Table 1. In total, 3434 (63.4%) participants reported regularly practicing a sport. Participants had a mean age of 25.5 years (SD = 1.26), 3121 (57.6%) were French-speakers and 2293 (42.4%) German-speakers. Participants who practiced a sport had a lower mean BMI of 23.9 kg/m^2^ (SD = 3.06) compared with those who did not practice a sport, 24.3 kg/m^2^ (SD = 4.22, *p* < 0.001) and a higher level of achieved education (60.9% of post-secondary school vs. 47.0%, *p* < 0.001).

### 3.2. Tobacco, Nicotine and Other Substance Use

Participants who practiced a sport reported a lower rate of cigarette smoking during the last 30 days (32.3%) compared with participants who did not practice a sport (44.6%), especially with lower rates of daily smokers (14.8% vs. 30.2%, *p* < 0.001). The proportion of occasionally and weekly snus users was higher in the group of participants practicing a sport (9.6% and 5.4%) compared with participants not practicing a sport (6.6% and 3.4%, *p* < 0.001). The same trend was observed among snuff users but without any statistically significant difference. In total 114 participants practicing a sport reported to be occasional vapers (3.3%) and 38 daily vapers (1.1%) compared with 115 (5.8%) and 31 (1.6%), respectively, among participants not practicing a sport (*p* < 0.001). Participants who practiced a sport were more likely to report an episode of binge drinking once a month or more (39.3%) compared with participants who did not practice a sport (36.8%, *p* < 0.001). On the contrary, risky alcohol use (more than or equal to 21 drinks per week) was more important among participants not practicing a sport, respectively 6.8% and 8.6% (*p* = 0.017). Participants who practiced a sport were also less likely to use cannabis (28.6% and 33.4%, *p* < 0.001) and illicit drugs (11.8% and 16.9%, *p* < 0.001). The association between tobacco or nicotine use and sport are shown in Table 2. Participants who practiced a sport were 41% less likely to smoke cigarettes (unadjusted, OR = 0.59, 95% CI = 0.53–0.66), with small changes after socio-demographic and BMI adjustment (model 1, OR = 0.63, 95% CI = 0.56–0.70) and substance use adjustment (model 2, OR = 0.68, 95% CI = 0.60–0.77). On the contrary, participants who practiced a sport were 57% more likely to use snus (unadjusted, OR = 1.57, 95% CI = 1.32–1.87) and the association was even stronger with multivariable adjustment (model 2, OR = 1.73, 95% CI = 1.44–2.07). For snuff use, results were only significant in the multivariable-adjusted model with participants in sport activity being 19% more likely to use snuff (model 2, OR = 1.19, 95% CI = 1.03–1.38). The inverse trend was observed for vaping. Participants in sport were 42% less likely to vape (unadjusted, OR = 0.58, 95% CI = 0.46–0.74, model 1, OR = 0.63, 95% CI = 0.50–0.80 and model 2, OR = 0.71, 95% CI = 0.55–0.90).

### 3.3. Type of Sport (Team vs. Individual) and Tobacco/Nicotine Use

Among participants who practiced a sport, 1016 (29.6%) participated in a team sport and 2418 (70.4%) participated in an individual sport (Table 3). The proportion of cigarette smokers did not statistically significantly differ between team-sport participants and individual-sport participants. We found a higher occasional (12.3%) and weekly (7.3%) snus use among team-sport participants compared with individual-sport participants (respectively 8.4% and 4.6%, *p* < 0.001). The same trend was observed for snuff use, with team-sport participants having a higher rate of occasional (21.4%) and weekly (2.3%) snuff use compared with individual-sport participants (15.5% and 2.4%, *p* < 0.001). On the contrary, we found an inverse trend for vaping, with more daily users among participants who practiced an individual sport (1.2%) than those who practiced a team sport (0.8%) but without statistical significance (*p* = 0.093). Logistic regression (Table 4) showed a statistically significant association between snus use and type of sport, with individual-sport participants being 39% less likely to use snus compared with team-sport participants (unadjusted, OR = 0.61, 95% CI = 0.50–0.74). The estimate did not change after adjustment for socio-demographic, BMI and other substance use (model 1, OR = 0.64, 95% CI = 0.53–0.79 and model 2, OR = 0.63, 95% CI = 0.51–0.77). Similarly, individual-sport participants were 30% less likely to use snuff compared with team-sport participants (unadjusted, OR = 0.70, 95% CI = 0.59–0.84) with no significant change after multivariable adjustment (model 1, OR = 0.74, 95% CI = 0.62–0.89 and model 2, OR = 0.73, 95% CI = 0.61–0.88). We found an inverse relationship regarding vaping, with individual-sport participants being 54% more likely to vape compared with team-sport participants in unadjusted model (unadjusted, OR = 1.54, 95% CI = 1.04–2.28, model 1, OR = 1.50, 95% CI = 1.01–2.23 and model 2, OR = 1.54, 95% CI = 1.03–2.30). There was no significant association between cigarette smoking and type of sport (individual sport vs. team sport).

### 3.4. Type of Sport (Mitchell’s Classification) and Tobacco/Nicotine Use

Participants who practiced a sport were classified according to Mitchell’s classification (Supplementary Table S1) [26]. We did not find any statistically significant association between type of sport according to Mitchell’s classification and cigarette smoking, snuff use or vaping with chi^2^ test. The only significant association found was for snus use, with the highest use in the B2 group (24.6%), then A2 (18.4%) and C2 (17.0%) and the lowest in the B1 group (8.6%) (*p* = 0.015). The use details are shown in Supplementary Figure S1 and Table S2. Logistic regressions showed that participants from the C3 group (highest static and dynamic component) were 52% less likely to smoke cigarettes, with unadjusted model (unadjusted, OR = 0.48, 95% CI = 0.27–0.85, model 1, OR = 0.50, 95% CI = 0.28–0.90 and model 2, OR = 0.41, 95% CI = 0.22–0.78) than participants from the A1 group. Same trend could be observed in participants who practiced a sport from the most intensive group being 70% less likely to vape, with unadjusted model (unadjusted OR = 0.30, 95% CI = 0.10–0.92, model 1, OR = 0.33, 95% CI = 0.11–1.00 and model 2, OR = 0.31, 95% CI = 0.10–0.98). The association with snuff use was only significant in the B3 and C2 groups with B3 and C2 group participants being less likely to use snuff. The association was not significant for snus use.

### 3.5. Intensity of Sport (MET/Min per Week) and Tobacco/Nicotine Use

In total, 1671 (48.7%) participants practiced a high intensity sport (1350 MET/min per week or more), 994 (28.9%) medium (between 675 and 1350 MET/min per week) and 769 (22.4%) low (less than 675 MET/min per week). The proportion of occasional and daily cigarette smokers was the highest (19.8% and 18.0%) in the low intensity group and the lowest in the high intensity group (15.7% and 12.9%, *p* < 0.001). The associations between snus, snuff, vaping and intensity of sport were not statistically significant. Details are shown in Supplementary Table S3. Logistic regression showed that participants who practiced a high intensity sport were 34% less likely to smoke cigarettes (OR = 0.66, 95% CI = 0.55–0.79). There was no statistically significant association between snus or snuff use and intensity of the sport.

## 4. Discussion

### 4.1. Major Findings

The aim of this study was to analyze the association between tobacco or nicotine use and intensity and type of sport. It is important to point out that the studied population was mostly athletic with 63.4% of the participants reporting regularly practicing a sport but slightly lower than the prevalence (70%) reported in Switzerland in 2020 among young men 25–34 years old [13]. Most participants practiced an individual sport (70.4%). Our findings suggest that people who practice a sport are less likely to smoke cigarettes or vape but are more likely to use snus. Furthermore, according to the type of sport, people who practice an individual sport are less likely to use snus or snuff but are more likely to vape, compared with those who practice a team sport. Results about the type of sport with Mitchell’s Classification did not show any progressive pattern according to the static or dynamic component of each sport. However, the intensity of a sport indicated an association with cigarette smoking: people who practiced a high intensity sport were less likely to smoke cigarettes, without any significant association for snus, snuff and vaping.

### 4.2. Comparison of Our Findings with Those Reported in the Literature

Our study suggests a negative association between sport and cigarette smoking, with people practicing a sport being less likely to smoke cigarettes but a positive association between sport and snus use. In the systematic review from Kaczynski et al. [17], the same negative association between physical activity and smoking was found, namely, people who practiced a sport were less likely to smoke. Compared to the literature [22,23], our study found the same results in Switzerland than in Finland and Norway according to the type of sport and snus use, namely, snus prevalence is higher among those who practice a team sport. In Canada, a positive association between team sport and smokeless tobacco has also been demonstrated among adolescents [32]. In a Finnish study [22] which included male participants during their first day of military service, the same association between sport and tobacco use was found. Furthermore, they found that snus use was associated with a higher sport intensity, which was not the case in our study. This could be explained by the higher prevalence of snus use in Scandinavian countries, 14.9% of snus use (exclusive or dual use) reported in this Finnish study compared to 13.2% in our study. In a longitudinal study performed among American adolescents [21] type of sport was also associated with tobacco use. Compared with our study, which classified sports into team and individual sports, they classified sport activities according to their association (negatively or positively associated with smoking) with different patterns of tobacco use according to the type of sport. Regarding sport intensity, in a Cypriot study which included adolescents and young adults, results were similar to ours: young people participating in high intensity activities were less likely to be smokers [15].

### 4.3. Interpretation of Our Findings

We found that participants who practiced a sport were less likely to be smokers. An explanation is that people who regularly practice a sport usually have a better lifestyle and consume fewer tobacco products and other related substances. Young people who practice a sport usually have a higher education level and it is known that education can influence smoking habits. However, as the association between tobacco or nicotine consumption and sport did not change in adjusted models for covariate (including highest achieved education), there must be other explanations. The association between intensity of sport and smoking could also be interpreted reverse: smokers tend to practice less high intensity sport because of lifestyle or lung capacity differences. On the contrary, people who practice a sport are more likely to use snus and people who practice a team sport are more likely to use snus and snuff. This could be explained by cultural rituals, for example a higher snus use in some environments such as ice hockey [22], or an influence coming from the Nordic countries. These traditions could also have an influence on sport adherence, inciting young people to consume tobacco or nicotine to be part of the group and therefore encouraging the addiction, bringing young people to practice a sport to consume tobacco or nicotine. Another explanation could be that athletes consume smokeless tobacco to enhance their performance, however, evidence for the effect of smokeless tobacco on performance is weak [33]. Furthermore, smokeless tobacco consumption is a risk factor for cancer, especially pancreas cancer, fatal myocardial infarction and other diseases [3]. Marketing could also play a role. In Switzerland, advertisement for tobacco products and vaping is not prohibited and could influence use of tobacco products. In our study, people who practiced a sport were less likely to vape. The young population is exposed to vaping through online advertisement [5,6] and sponsorship in music festivals and social events. Thus, young people who practice a sport might be less exposed to and, consequently, less influenced by these ads. Peer influence might also play an important role in tobacco and nicotine products use. Although the smoking behavior of the coaches does not seem to influence the smoking habits of youth, in a Swedish study including female athletes [23], the smoking habits of teammates or friends could play a role. Furthermore, the importance of collective events related to a sport could explain the higher snus and snuff use in team sports.

### 4.4. Strengths and Limitations

We have to acknowledge some limitations in our study. Although assessment was independent of army procedures and military service, the mandatory recruitment procedures were used to enroll participants. Nevertheless, this means that women or inhabitants of Switzerland without Swiss nationality were not included. This limits the generalizability of our findings. However, the study participants represent a good and important sample of young Swiss men, recruitment being mandatory in Switzerland (except for severe medical conditions). All data were self-reported which could induce an over-estimation of positive behaviors (such as physical activity [34]) and under-estimation of negative ones (such as tobacco use), due to a social desirability bias. We assumed that the most frequently played sport had the most influence on tobacco use. Finally, due to the cross-sectional design of the study, we were not able to determine the direction of the association and if there was a causal relationship.

This study has some strengths. We had a large sample and good measures of health behaviors, including numerous potential confounding factors that we could include in our models. We could characterize in detail the type of sport played and its association with tobacco or nicotine use. We also collected detailed information on type of tobacco or nicotine products used.

## 5. Conclusions

In conclusion, youth who practice a sport are less likely to smoke cigarettes or vape but are more likely to use snus. Our findings suggest that the type and intensity of sport are associated with tobacco use. Youth who practice an individual sport are less likely to use snus or snuff and more likely to vape compared with a team sport. Youth practicing a high intensity sport are less likely to smoke cigarettes compared with a low intensity sport. The findings of this study could help to better target tobacco use prevention in Switzerland, not only for cigarettes but also for smokeless products in team sports. To our knowledge, tobacco use prevention in Switzerland is mainly directed at smoking but should also target the use of smokeless nicotine products in sport environment. As our study only involved Swiss men, the results cannot be extrapolated to Swiss women but similar findings could be expected. Further studies should be done to determine which sports are most associated with tobacco use in order to better target prevention.

## Figures and Tables

**Table 1:** Sample characteristics of participants (N = 5414).

	No Sport (N = 1980, 36.6%)		Sport (N = 3434, 63.4%)		Total (N = 5414)		*p*-Value
Age, Mean (SD)	25.6	1.28	25.4	1.24	25.5	1.26	<0.001
Highest achieved education, N (%)							<0.001
primary schooling	106	5.4	83	2.4	189	3.5	
vocational schooling	944	47.7	1261	36.7	2205	40.7	
post-secondary school	930	47.0	2090	60.9	3020	55.8	
BMI continuous, Mean (SD)	24.3	4.22	23.9	3.06	24.1	3.53	<0.001
Linguistic region, N (%)							0.064
French	1109	56.0	2012	58.6	3121	57.6	
German	871	44.0	1422	41.4	2293	42.4	
Cigarette smoking, N (%)							<0.001
no use	1097	55.4	2324	67.7	3421	63.2	
occasional	286	14.4	602	17.5	888	16.4	
daily	597	30.2	508	14.8	1105	20.4	
Snus use, N (%)							<0.001
no use	1781	90.0	2921	85.1	4702	86.9	
occasional	131	6.6	328	9.6	459	8.5	
weekly	68	3.4	185	5.4	253	4.7	
Snuff use, N (%)							0.314
no use	1625	82.1	2763	80.5	4388	81.1	
occasional	316	16.0	591	17.2	907	16.8	
weekly	39	2.0	80	2.3	119	2.2	
Vaping, N (%)							<0.001
no use	1834	92.6	3282	95.6	5116	94.5	
occasional	115	5.8	114	3.3	229	4.2	
daily	31	1.6	38	1.1	69	1.3	
Alcohol use: binge drinking, N (%)							<0.001
never	487	24.6	669	19.5	1156	21.4	
< once a month	764	38.6	1417	41.3	2181	40.3	
≥ once a month	729	36.8	1348	39.3	2077	38.4	
Alcohol at risk use, N (%)							0.017
not at risk	1810	91.4	3200	93.2	5010	92.5	
at risk	170	8.6	234	6.8	404	7.5	
Cannabis use, N (%)							<0.001
no use	1318	66.6	2450	71.4	3768	69.6	
occasional	414	20.9	774	22.5	1188	21.9	
more than weekly	248	12.5	210	6.1	458	8.5	
Drugs use, N (%)							<0.001
no use	1646	83.1	3030	88.2	4676	86.4	
use	334	16.9	404	11.8	738	13.6	

**Table 2:** Association between tobacco or nicotine use and sport (vs no sport).

	Unadjusted Model	*p*-Value	Model 1	*p*-Value	Model 2	*p*-Value
	OR (95% CI)		OR (95% CI)		OR (95% CI)	
Cigarette smoking	0.59(0.53–0.66)	<0.001	0.63(0.56–0.70)	<0.001	0.68(0.60–0.77)	<0.001
Snus use	1.57(1.32–1.87)	<0.001	1.63(1.36–1.94)	<0.001	1.73(1.44–2.07)	<0.001
Snuff use	1.11(0.96–1.28)	0.145	1.15(0.99–1.33)	0.065	1.19(1.03–1.38)	0.022
Vaping	0.58(0.46–0.74)	<0.001	0.63(0.50–0.80)	<0.001	0.71(0.55–0.90)	0.005

Note. Model 1: Adjusted for sociodemographics and body mass index. Model 2: adjusted for sociodemographics, body mass index and substance use.

**Table 3:** Association between tobacco or nicotine use and type of sport (team/individual) (N = 3434).

	Team (N = 1016, 29.6%)		Individual (N = 2418, 70.4%)		Total (N = 3434)		*p*-Value
	N	%	N	%	N	%	
Cigarette smoking							0.532
no use	691	68.0	1633	67.5	2324	67.7	
occasional	168	16.5	434	18.0	602	17.5	
daily	157	15.5	351	14.5	508	14.8	
Snus use							<0.001
no use	817	80.4	2104	87.0	2921	85.1	
occasional	125	12.3	203	8.4	328	9.6	
weekly	74	7.3	111	4.6	185	5.4	
Snuff use							<0.001
no use	776	76.4	1987	82.2	2763	80.5	
occasional	217	21.4	374	15.5	591	17.2	
weekly	23	2.3	57	2.4	80	2.3	
Vaping							0.093
no use	983	96.8	2299	95.1	3282	95.6	
occasional	25	2.5	89	3.7	114	3.3	
daily	8	0.8	30	1.2	38	1.1	

**Table 4:** Association between tobacco or nicotine use and individual sport (vs team sport).

	Unadjusted Model	*p*-Value	Model 1	*p*-Value	Model 2	*p*-Value
	OR (95% CI)		OR (95% CI)		OR (95% CI)	
Cigarette smoking	1.02 (0.87–1.20)	0.785	1.03 (0.88–1.21)	0.720	1.01 (0.85–1.20)	0.925
Snus use	0.61 (0.50–0.74)	<0.001	0.64 (0.53–0.79)	<0.001	0.63 (0.51–0.77)	<0.001
Snuff use	0.70 (0.59–0.84)	<0.001	0.74 (0.62–0.89)	0.001	0.73 (0.61–0.88)	0.001
Vaping	1.54 (1.04–2.28)	0.031	1.50 (1.01–2.23)	0.044	1.54 (1.03–2.30)	0.036

Note. Model 1: Adjusted for sociodemographics and body mass index. Model 2: adjusted for sociodemographics, body mass index and substance use.

## Supplementary Materials

The following are available online at https://www.mdpi.com/1660-4601/17/22/8299/s1, Table S1: Type of sports practiced by the participants of the study according to Mitchell’s classification (N = 3434) [26], Table S2: Association between tobacco or nicotine use and A1 group of Mitchell’s classification (vs other groups) [26], Table S3: Association between tobacco or nicotine use and low intensity of sport (vs medium and high intensity of sport), Figure S1: Association between type of sport (according to Mitchell’s Classification) and tobacco and nicotine use (cigarette, snus, snuff, vaping) [26].
