# Supplementary material for: The Association between Type and Intensity of Sport and Tobacco or Nicotine Use—A Cross-Sectional Study among Young Swiss Men
Source: Int J Environ Res Public Health. 2020 Nov 10;17(22):8299. doi: 10.3390/ijerph17228299 (PMC7696790; doi:10.3390/ijerph17228299)

International Journal of Environmental Research and Public Health

MDPI

Supplementary Files

Table S1. Type of sports practiced by the participants of the study according to Mitchell's classification (N=3434) [26].

**III. High Static Component (>50%MVC)**	Circus, Kitesurf/Windsurf/ Paddle, Paragliding/Skydiving, Martial Art, Gymnastic, Sailing, Sport climbing, N=302	Fitness/Bodybuilding (musculation), Downhill bicycling, Skiing/Snowboarding/Telemark, Skateboarding, Dancing, Fighting sport, N=785	Speed Skating, Bicycling, Boxing, Rowing, Kayaking, Triathlon, N=346
**II. Moderate Static Component (20-50%MVC)**	Motorcycling, Juggling, Archery, Auto racing/Karting, Diving, Horse riding (Equestrian), N=38	Running (sprint), Roller (Figure skating), Athletics, Parkour, Surfing, Dog sport, American football/Rugby, N=57	Radball, Fitness (cardio, endurance), Crossfit, Alpinism/Mountaineering/Ski touring, Fistball, Basketball, Lacrosse, Cross-country-skiing, Tchoukball, Water polo, Ice hockey, Swimming, Running, Kin-ball N=820
**I. Low Static Component (<20%MVC)**	Yoga/Pilatus, E-sport, Bowling/Curling/Petanque, Fishing, Billiards, Golf, Shooting sports, Darts, N=56	Paintball, Hornuss, Baseball, Table tennis, Fencing, Volleyball/Beach volley, Walking, N=140	Running (long distance), Squash, Orienteering, Tennis, Badminton, Unihockey/Fieldhockey/Street-hockey/Skater-hockey, Football (player, referee), N=890
	**A. Low Dynamic Component (<40% Max O2)**	**B. Moderate Dynamic Component (40-70% Max O2)**	**C. High Dynamic Component (>70% Max O2)**

Table S2. Association between tobacco or nicotine use and A1 group of Mitchell's classification (vs other groups) [26].

	**Unadjusted Model**	**P-value**	**Model 1**	**P-value**	**Model 2**	**P-value**
	**OR (95% CI)**		**OR (95% CI)**		**OR (95% CI)**	
Cigarette smoking						
A2	0.81(0.35-1.87)	0.619	0.79(0.34-1.82)	0.576	0.66(0.26-1.68)	0.384
A3	0.56(0.31-1.00)	0.051	0.59(0.33-1.06)	0.076	0.45(0.24-0.84)	0.013
B1	0.53(0.28-1.01)	0.052	0.56(0.29-1.06)	0.073	0.52(0.26-1.05)	0.070
B2	0.53(0.24-1.14)	0.105	0.53(0.24-1.15)	0.110	0.38(0.16-0.90)	0.028
B3	0.68(0.39-1.17)	0.167	0.70(0.41-1.22)	0.208	0.61(0.33-1.10)	0.101
C1	0.62(0.36-1.07)	0.083	0.65(0.38-1.13)	0.125	0.61(0.33-1.10)	0.102
C2	0.53(0.31-0.92)	0.025	0.57(0.33-0.98)	0.043	0.53(0.29-0.97)	0.039

C3	0.48(0.27-0.85)	0.012	0.50(0.28-0.90)	0.020	0.41(0.22-0.78)	0.006
Snus use						
A2	1.58(0.51-4.94)	0.431	1.70(0.54-5.41)	0.366	1.73(0.54-5.53)	0.355
A3	1.04(0.44-2.45)	0.932	1.13(0.47-2.69)	0.786	1.05(0.44-2.53)	0.906
B1	0.66(0.24-1.76)	0.404	0.65(0.24-1.78)	0.405	0.66(0.24-1.81)	0.423
B2	2.28(0.84-6.17)	0.105	2.11(0.77-5.77)	0.147	2.12(0.77-5.86)	0.148
B3	1.01(0.45-2.29)	0.981	0.99(0.43-2.26)	0.979	0.95(0.41-2.18)	0.896
C1	1.41(0.63-3.17)	0.409	1.42(0.62-3.21)	0.407	1.41(0.62-3.22)	0.416
C2	1.43(0.63-3.22)	0.389	1.45(0.64-3.29)	0.379	1.45(0.64-3.32)	0.376
C3	1.10(0.47-2.57)	0.825	1.14(0.48-2.70)	0.758	1.12(0.47-2.65)	0.803
Snuff use						
A2	0.56(0.21-1.54)	0.265	0.58(0.21-1.63)	0.305	0.61(0.22-1.71)	0.348
A3	0.69(0.36-1.30)	0.249	0.74(0.38-1.42)	0.365	0.70(0.36-1.36)	0.291
B1	0.60(0.29-1.22)	0.158	0.59(0.28-1.22)	0.156	0.60(0.29-1.25)	0.174
B2	0.67(0.28-1.58)	0.356	0.59(0.25-1.41)	0.236	0.61(0.25-1.48)	0.272
B3	0.50(0.27-0.92)	0.026	0.48(0.26-0.89)	0.019	0.45(0.24-0.85)	0.014
C1	0.72(0.39-1.30)	0.274	0.70(0.38-1.30)	0.262	0.70(0.37-1.29)	0.251
C2	0.52(0.29-0.96)	0.037	0.51(0.28-0.96)	0.035	0.51(0.27-0.95)	0.034
C3	0.67(0.35-1.26)	0.214	0.69(0.36-1.31)	0.256	0.68(0.35-1.30)	0.240
Vaping						
A2	0.28(0.03-2.46)	0.248	0.25(0.03-2.28)	0.221	0.24(0.03-2.24)	0.211
A3	0.57(0.20-1.63)	0.294	0.60(0.21-1.72)	0.337	0.57(0.19-1.67)	0.303
B1	0.07(0.01-0.64)	0.018	0.08(0.01-0.68)	0.021	0.07(0.01-0.64)	0.018
B2	0.37(0.69-2.00)	0.248	0.42(0.08-2.28)	0.313	0.43(0.08-2.42)	0.341
B3	0.61(0.23-1.59)	0.310	0.64(0.24-1.70)	0.368	0.64(0.23-1.74)	0.381
C1	0.43(0.16-1.14)	0.090	0.46(0.17-1.23)	0.120	0.47(0.17-1.28)	0.139
C2	0.48(0.18-1.28)	0.143	0.51(0.19-1.38)	0.185	0.53(0.19-1.47)	0.224
C3	0.30(0.10-0.92)	0.036	0.33(0.11-1.00)	0.050	0.31(0.10-0.98)	0.046

Note. Model 1: Adjusted for sociodemographics and body mass index. Model 2: adjusted for sociodemographics, body mass index and substance use.

Table S3. Association between tobacco or nicotine use and low intensity of sport (vs medium and high intensity of sport).

	**Unadjusted Model**	**P-value**	**Model 1**	**P-value**	**Model 2**	**P-value**
	**OR (95% CI)**		**OR (95% CI)**		**OR (95% CI)**	
Cigarette smoking						
675-1350 MET/min*week	0.86(0.71-1.05)	0.139	0.88(0.72-1.07)	0.205	0.87(0.70-1.07)	0.196
>1350 MET/min*week	0.66(0.55-0.79)	<0.001	0.67(0.56-0.80)	<0.001	0.63(0.52-0.78)	<0.001
Snus use						
675-1350 MET/min*week	0.86(0.66-1.12)	0.268	0.85(0.65-1.11)	0.227	0.83(0.63-1.10)	0.193
>1350 MET/min*week	1.02(0.81-1.29)	0.862	1.04(0.82-1.32)	0.764	1.06(0.83-1.35)	0.660
Snuff use						
675-1350 MET/min*week	1.06(0.84-1.35)	0.608	1.05(1.83-1.34)	0.668	1.04(0.82-1.33)	0.738

>1350 MET/min*week	0.95(0.77-1.18)	0.647	0.96(0.77-1.20)	0.731	0.98(0.78-1.23)	0.862
Vaping						
675-1350 MET/min*week	0.60(0.39-0.95)	0.028	0.63(0.40-0.99)	0.043	0.62(0.39-0.99)	0.044
>1350 MET/min*week	0.71(0.49-1.05)	0.085	0.70(0.47-1.03)	0.068	0.72(0.48-1.06)	0.095

Note. Model 1: Adjusted for sociodemographics and body mass index. Model 2: adjusted for sociodemographics, body mass index and substance use.

Figure S1. Association between type of sport (according to Mitchell's Classification) and tobacco and nicotine use (cigarette, snus, snuff, vaping) [26].

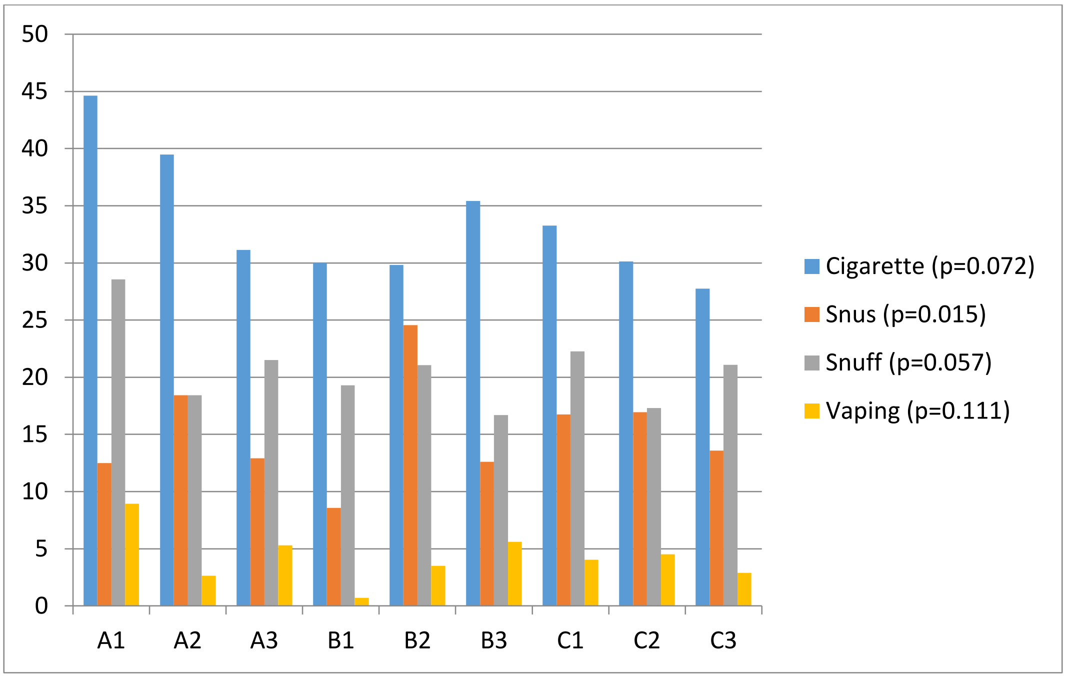

Supplement: Supplementary file 1 [file ijerph-17-08299-s001.pdf]
